# The role of EGFR‐TKIs as adjuvant therapy in EGFR mutation‐positive early‐stage NSCLC: A meta‐analysis

**DOI:** 10.1111/1759-7714.13874

**Published:** 2021-03-04

**Authors:** Chutong Lin, Fengling Hu, Hongling Chu, Peng Ren, Shanwu Ma, Jingdi Wang, Jie Bai, Xuan Han, Shaohua Ma

**Affiliations:** ^1^ Department of Thoracic Surgery Peking University Third Hospital Beijing China; ^2^ Research Center of Clinical Epidemiology Peking University Third Hospital Beijing China

**Keywords:** adjuvant treatment, epidermal growth factor receptor tyrosine kinase inhibitors, nonsmall‐cell lung cancer, survival, targeted therapy

## Abstract

**Background:**

The role of adjuvant epidermal growth factor receptor tyrosine kinase inhibitors (EGFR‐TKIs) is not clear in early‐stage nonsmall‐cell lung cancer (NSCLC) patients. This meta‐analysis aims to compare the efficacy and safety of EGFR‐TKIs as adjuvant therapy with chemotherapy or placebo in NSCLC patients harboring EGFR mutations.

**Patients and Methods:**

Pubmed, Embase, and Cochrane databases were searched for randomized controlled trials. The hazard ratio (HR) of disease‐free survival (DFS) and overall survival (OS) as well as the risk ratio (RR) of severe adverse events were merged.

**Results:**

Seven articles from five studies from 1843 records, a total of 1227 patients, were included in the analysis. The HR for DFS was 0.38 (95% confidence interval [CI] 0.22–0.63), in favor of EGFR‐TKIs. However, no significant benefit of OS was seen (HR = 0.61, 95% CI 0.31–1.22). Treatment benefit was more pronounced in patients with advanced disease stage and longer duration of medication, EGFR exon 19 deletion mutation, and treatment with third‐generation EGFR‐TKIs. Adjuvant targeted therapy may cause few adverse events compared with chemotherapy (RR = 0.28, 95% CI 0.09–0.94). The possibility of severe adverse events for the first‐generation drugs was significantly lower than for third‐generation drugs.

**Conclusion:**

In EGFR mutation‐positive patients with stage IB–IIIA NSCLC, compared with adjuvant chemotherapy or placebo, adjuvant EGFR‐TKIs should effectively improve the patient's DFS, but not effectively improve OS. Disease stage, treatment duration, mutation types, and therapeutic drugs could affect the degree of benefit. Adjuvant EGFR‐TKIs had more favorable tolerability than chemotherapy, especially with the usage of first‐generation drugs.

## Graphical abstract

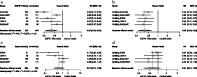

## INTRODUCTION

Lung cancer is one of the most common malignant tumors, ranked second in incidence among all tumors following nonmelanoma skin cancer.^1^ With a heavy burden worldwide, lung cancer is the leading cause of tumor‐related deaths and disability‐adjusted life years in males and the second most common cause in females. Nonsmall‐cell lung cancer (NSCLC) is the major subtype of lung cancer, accounting for about 85% of all cases.^2^ Most patients have advanced disease when diagnosed, and about 30% of NSCLC patients are candidates for radical resection.^3^, ^4^

Postoperative adjuvant treatment is recommended for candidate patients (those with stage IIB–IIIA disease or partly stage IB and IIA disease) with NSCLC who have undergone radical surgical resection to reduce the risk of recurrence and metastasis.^5^ Platinum‐based doublet chemotherapy is the current standard for adjuvant treatment with a 5‐year overall survival (OS) improvement of 5.4% when compared with placebo.^6^, ^7^ Despite some advancement in safety and tolerability, limited improvement was seen in efficacy from adjuvant chemotherapy.^8^, ^9^ Unmet clinical need remains in the adjuvant setting.

The last decade has witnessed the evolving role of epidermal growth factor receptor tyrosine kinase inhibitors (EGFR‐TKIs) as the first‐line treatment for EGFR‐mutant advanced NSCLC for their favorable efficacy and safety.^10^, ^11^, ^12^, ^13^ The biomarker‐response relationship has also been clarified in the IPASS study, which demonstrated EGFR mutation is the strongest predictor for the therapeutic effect of EGFR‐TKIs.^14^ Targeted therapy with imatinib has been used as postoperative adjuvant treatment for gastrointestinal tumors.^15^ Therefore, investigation has begun into the role of adjuvant EGFR‐TKIs in EGFR mutation‐positive NSCLC patients who have undergone radical surgery.^16^

Randomized controlled trials and meta‐analysis found adjuvant treatment with EGFR‐TKIs after surgery may improve the disease‐free survival (DFS) of patients with early‐stage NSCLC.^17^, ^18^, ^19^, ^20^ However, no significant improvement in OS has been determined due to the immature OS data. In addition, the sample size in most studies was relatively small, and thus not powerful enough to detect the OS benefit from adjuvant EGFR‐TKIs. Questions remain in terms of the timing of adjuvant EGFR‐TKIs, the duration of medication, the preferred drugs, and preferred gene mutation type owing to the heterogeneous results of different studies. Recently, the ADJUVANT study updated its OS data,^21^ and the preliminary data of ADAURA has also been published.^16^ Therefore, we conducted a meta‐analysis of the randomized controlled trials to compare the efficacy between adjuvant EGFR‐TKIs and placebo/chemotherapy in EGFR mutation‐positive NSCLC patients. Potential influencing factors were also evaluated.

## METHODS

This analysis was presented according to the preferred reporting items for systematic reviews and meta‐analyses statement (PRISMA) and was registered with the PROSPERO register of systematic reviews (registration number: CRD42020213849).

### Literature search strategies

We searched Pubmed, Embase, and the Cochrane Central Library from 1 January 2000 to 20 September 2020 using the keywords “carcinoma, non‐small‐cell lung” and its synonyms, epidermal growth factor receptor, resectable, operable, adjuvant, postoperative, and survival. The search was limited to clinical trials. Abstracts of academic conferences including the American Society of Clinical Oncology Annual meeting, the World Conference on Lung Cancer, the European Society for Medical Oncology, and registered clinical trials were also included to identify relevant studies.

### Study eligibility and selection

Clinical trials that met the following criteria were included: (a) randomized controlled trials in NSCLC patients with EGFR‐mutant, stage IB–IIIA disease that underwent radical resection; (b) random assignment of participants to treatment with postoperative EGFR‐TKI or chemotherapy/placebo; and (c) reporting of DFS and OS. Exclusion criteria were (a) studies in which adjuvant immunotherapy was evaluated; and (b) studies with a sample size less than 50. Duplicate records and republished studies, reviews, meta‐analysis, case reports, and non‐English reports were also excluded from the analysis.

### Data extraction

The following information was recorded for each study: first author, year of publication, name of the study, sample size, gender, average age, disease stage, median follow‐up duration, number of cases in intervention and control groups, treatment strategy, number of adverse events (≥ grade 3),the hazard ratio (HR) value of DFS and OS, the subtype of EGFR mutation, and the median duration of treatment. Data extraction was done independently by two authors and discrepancies were resolved by consensus that included a third author.

### Quality assessment of the included studies

Two authors independently evaluated the quality of the included studies based on the Cochrane Collaboration's Tool for assessing risk of bias in the *Cochrane Handbook for Systematic Reviews of Interventions*, which includes selection bias, implementation bias, measurement bias, loss to follow‐up bias, publication bias, and other biases. Differences and disagreements were resolved by discussion with introduction of a third author.

### Statistical analysis

The data analysis of this study was done using R, version 3.6.2 (R Core Team). Log‐transformed HR and standard error values combined with the generic inverse variance method were used to evaluate HR in DFS and OS. For adverse events analysis, we collected the number of events and participants in each study to estimate the RR. Power analysis was performed using an alpha error probability of 0.05. We applied the *I* ^*2*^ test and the Q test for heterogeneity estimation.^22^ *I* ^*2*^ > 50% or Q test *p* < 0.1 were considered significant heterogeneity and the random effects model was used. Otherwise, the fixed‐effects model was used. Publication bias was evaluated by Egger's test.

## RESULTS

### Characteristics of included studies

**FIGURE 1:**
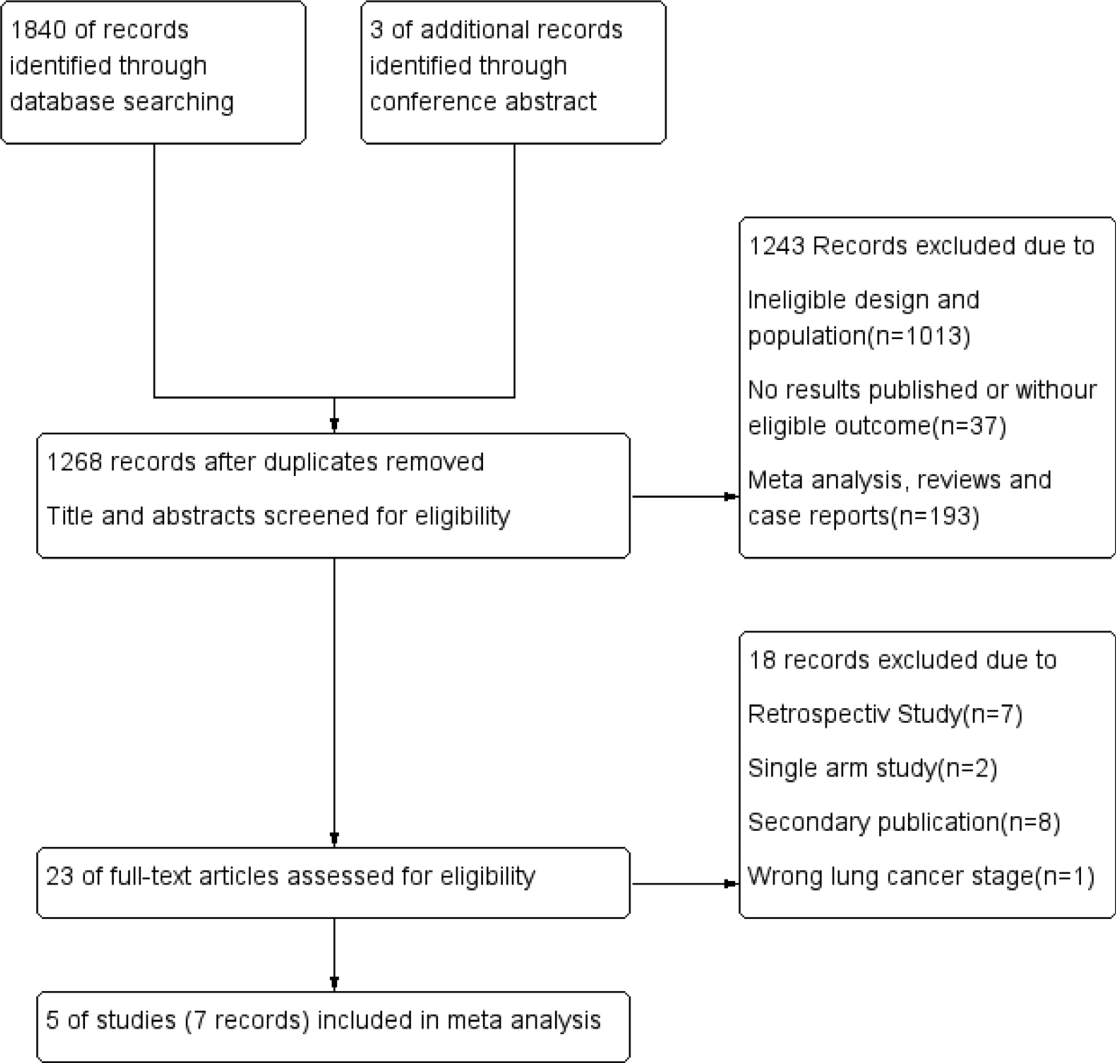
Flow chart of selection strategy

The selection strategy and reasons for exclusion are shown in Figure 1. A total of 1843 records were identified. After screening, seven articles of five randomized controlled trials were considered eligible for the meta‐analysis,^16^, ^21^, ^23^, ^24^, ^25^, ^26^, ^27^ consisting of a total of 1227 patients with EGFR mutation (633 patients in the experimental group and 594 patients in the control group). While some of the patients in the RADIANT study were without EGFR mutation, only patients with EGFR mutation positive (161 cases of 973 cases) were taken into the analysis.

Characteristics of included trials are presented in Table 1. The ADAURA study is the largest study, with 682 patients. The EVAN study and Li et al.'s study mainly focused on patients with stage IIIA disease. The ADJUVANT study excluded stage IB patients while 47% patients had stage IB disease in the RADIANT study. The ADAURA study evaluated the third‐generation EGFR‐TKI osimertinib, and the remaining studies evaluated first‐generation drugs. In the Li et al.'s study, the planned medication time was 6 months, and the median treatment duration was not reported. The planned medication time in the ADAURA study was 3 years, while the rest of study was 2 years. The median treatment duration was more than 20 months in most studies except for the study by Li et al. The ADAURA study only reported DFS data, so it was not included in the OS analysis.

**TABLE 1:** Characteristics of included trials

Trial	Sample size	Disease stage			Treatment				HR				EGFR‐TKIs	Median treatment duration (months)	Mutation subtype	
		IB	II	IIIA	Intervention	*N*	Control	*N*	DFS	95% CI	OS	95% CI			Exon 19	Exon 21
Roy 2020 (ADAURA)^16^	682	32.0%	34.0%	34.0%	(Chemo+) TKIs	339	(Chemo+) Placebo	343	0.20	0.14–0.30	—	—	Osimertinib	22.5	55.5%	44.5%
Zhong 2018 (ADJUVANT)^21^, ^26^	222	—	34.0%	64.4%	TKIs	111	Chemo	111	0.60	0.42–0.87	0.96	0.64–1.43	Gefitinib	21.9	51.8%	48.0%
Yue 2018 (EVAN)^25^	102	—	—	100.0%	TKIs	51	Chemo	51	0.27	0.14–0.53	0.17	0.05–0.58	Erlotinib	23.9	56.9%	42.2%
Li 2014^24^	60	—	—	100.0%	Chemo + TKIs	30	Chemo	30	0.37	0.16–0.85	0.37	0.12–1.11	Gefitinib	‐	33.3%	66.7%
Kelly 2015 (RADIANT)^23^, ^27^	161	47.0%	29.0%	22.0%	(Chemo+) TKIs	102	(Chemo+) Placebo	59	0.60	0.38–0.98	1.09	0.56–2.16	Erlotinib	21.2	41.0%	56.4%

### Study quality

**FIGURE 2:**
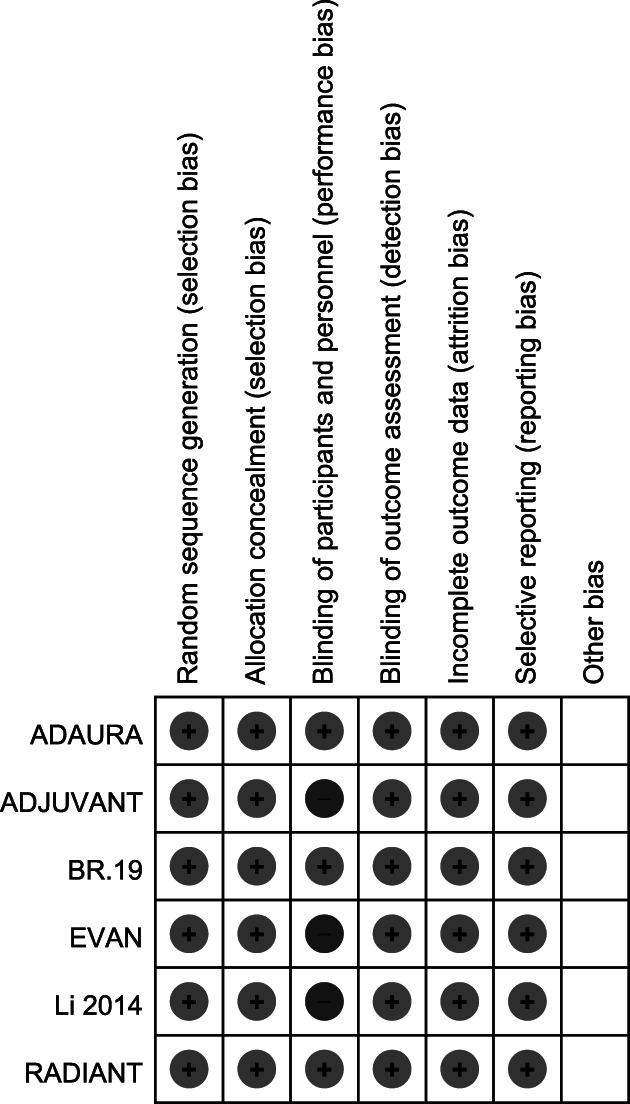
Assessment of study quality included in the meta‐analysis by Cochrane collaboration's tool for assessing risk of bias.Note: Low and high scores given for the seven parameters assessed represented by ‘+’ and ‘−’

We evaluated the quality of trials according to the Cochrane ‘Risk of bias’. As seen in Figure 2, two studies were at low risk of bias while three studies had one domain each marked as high risk (performance bias).

### DFS

**FIGURE 3:**
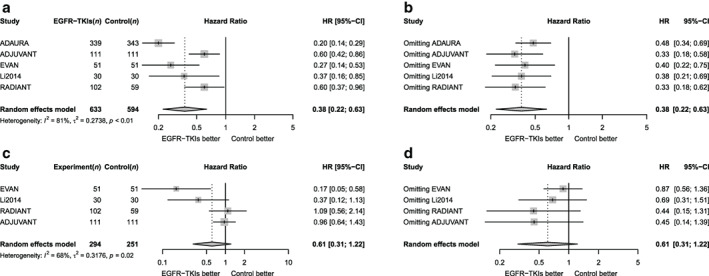
Forest plot for hazard ratio on DFS and OS. (a) Forest plot of hazard ratio of DFS. (b) Sensitivity analysis for DFS. (c) Forest plot of hazard ratio of OS. (d) Sensitivity analysis for OS

The random effects model was used for meta‐analysis because of strong heterogeneity among the five included studies (*I* ^*2*^ = 81%, *p* < 0.1) (Figure 3(a)). Compared with the control group, the risk of disease recurrence with adjuvant treatment with EGFR‐TKIs was reduced by 62% with statistical significance (HR = 0.38, 95% confidence interval [CI] 0.22–0.63). Egger's test revealed no evidence of publication bias (*p* = 0.89). In the sensitivity analysis, deletion of any research did not affect the results of this meta‐analysis, indicating the result of random effects was stable and reliable (Figure 3(b)).

### Subgroup analysis for DFS

Table 2 summarizes the subgroup analysis of DFS. Adjuvant EGFR‐TKIs were associated with a significantly increased DFS benefit in patients with stage IIIA disease, exon 19 deletion mutation, and those treated with third‐generation EGFR‐TKIs (all *p* < 0.01). Regardless of the presence of previous adjuvant chemotherapy, postoperative patients could benefit from adjuvant EGFR‐TKIs in terms of DFS to a similar degree (*p* = 0.21).

**TABLE 2:** Subgroup analysis of effect on DFS from EGFR‐TKI treatment

Category	Studies divided to subgroups	No. of patients		HR (95% CI)	Heterogeneity *I* ^*2*^	Intersubgrou*p* heterogeneity	
		Intervention	Control			*I* ^2^	*p* value
Disease stage						81%	<0.01
IIIA	EVAN, Li et al.	81	81	0.30 (0.18, 0.52)	0%		
IB‐IIIA	ADAURA, ADJUVANT, RADIANT	552	513	0.41 (0.20, 0.87)	90%		
Mutation type						80%	<0.01
Exon 19 deletion	ADAURA, ADJUVANT, Li et al.	258	255	0.22 (0.05, 0.95)	88%		
Exon 21 L858R	ADAURA, ADJUVANT, Li et al.	224	226	0.43 (0.27, 0.70)	47%		
Median treatment duration						86%	<0.01
<22.3 months	ADJUVANT, RADIANT	213	170	0.60 (0.45, 0.80)	0%		
≥22.3 months	ADAURA, EVAN	390	394	0.21 (0.15, 0.30)	0%		
Treatment of experimental arms						81%	<0.01
First‐generation EGFR TKI	ADJUVANT, EVAN, RADIANT, Li et al.	294	251	0.51 (0.40, 0.66)	42%		
Third‐generation EGFR TKI	ADAURA	339	343	0.20 (0.14, 0.29)	—		
Different comparation						81%	<0.01
vs. chemotherapy	ADJUVANT, EVAN	162	162	0.42(0.19, 0.93)	76%		
vs. placebo	ADAURA, Li et al., RADIANT	471	432	0.35(0.16, 0.76)	84%		
Previous adjuvant chemotherapy before EGFR‐T KI treatment						36%	0.21
Yes	ADAURA, Li et al.	235	235	0.20 (0.13, 0.30)	66%		
No	ADAURA	136	136	0.23 (0.13, 0.40)	—		

The risk of disease recurrence in patients who received adjuvant targeted therapy after surgery was greatly reduced, being only 42% of that with adjuvant chemotherapy (HR = 0.42, 95% CI 0.19–0.93) and 35% of that with placebo (HR = 0.35, 95% CI 0.16–0.76).

Li et al.'s study was excluded from subgroup analysis for medication duration. The median medication duration of the remaining four studies was extracted and the weighted average was 22.3 months based on the sample size. Taking 22.3 months as the cut‐off value, patients with longer medication duration (≥ 22.3 months) had more DFS (*p* < 0.01).

High heterogeneity was noted among the studies (*I* ^*2*^ = 81%; Figure 3(a)). After dividing all studies into two groups according to the median medication duration, the heterogeneity within the two groups was completely eliminated (*I* ^*2*^ = 0% in both groups), but the heterogeneity between the groups was significant (*I* ^*2*^ = 86%, *p* < 0.1). Therefore, the difference in the benefit of adjuvant EGFR‐TKIs for DFS among different studies may be largely attributed to the difference in treatment duration.

### Overall survival

Four studies were included in the OS analysis. The *I* ^*2*^ score was 68%, suggesting moderate heterogeneity, therefore the random effects model was used for meta‐analysis (Figure 3(c)).

The risk of all‐cause death was reduced by 39% in patients treated with adjuvant EGFR‐TKIs compared with those in the control group, but it was not statistically significant (HR = 0.61, 95% CI 0.31–1.22). The Egger's test suggested no significant evidence of publication bias (Egger's *p* = 0.15). In the sensitivity analysis, deleting any study did not affect the results of this analysis, indicating the calculation results of the random effects model were stable and reliable (Figure 3(d)).

### Subgroup analysis for OS

#### OS subgroup analysis of different disease stages

**FIGURE 4:**
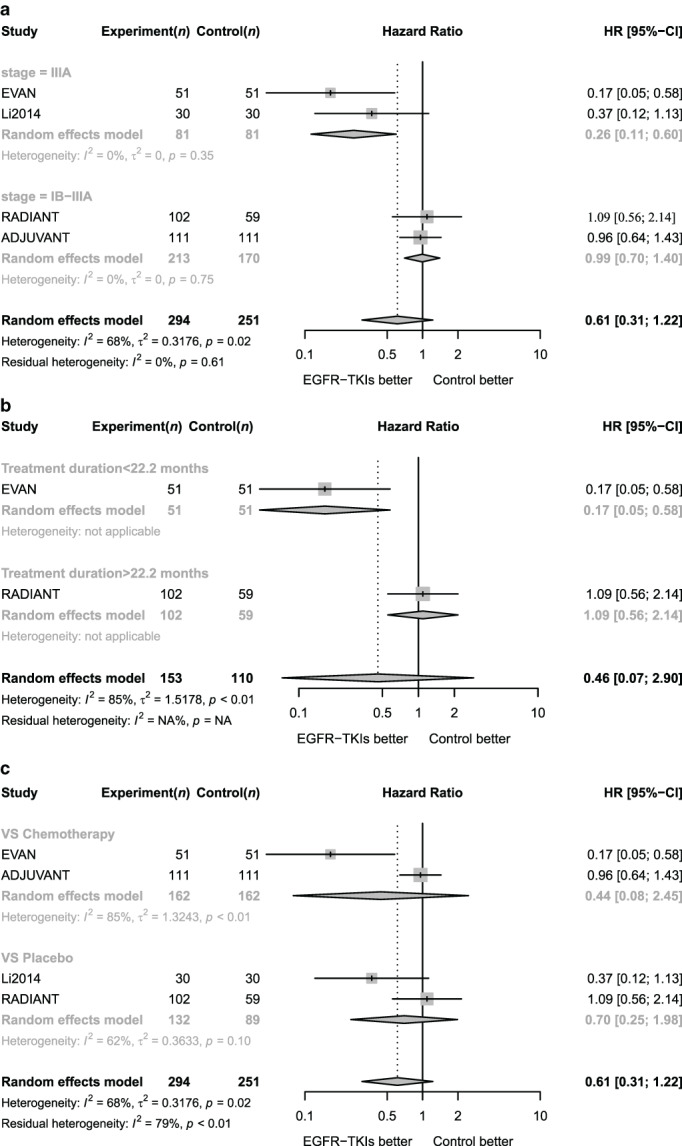
Subgroup analysis for OS for patients receiving adjuvant EGFR‐TKIs. (a) Forest plot of hazard ratio of OS for adjuvant EGFR TKI according to disease stages. (b) Forest plot of hazard ratio of OS for adjuvant EGFR TKI according to median treatment duration

Moderate heterogeneity was seen between the two groups according to disease stage (*I* ^*2*^ = 68%, *p* < 0.1; Figure 4(a)), suggesting patients with different disease stages were associated with varied survival benefits from treatment with EGFR‐TKIs. In patients with stage IIIA disease, the HR for OS was 0.26 (95% CI 0.11–0.60), which favored EGFR‐TKIs. No heterogeneity was seen with studies that incorporated mixed patients of stage IB–IIIA (*I* ^*2*^ = 0%). However, for these patients adjuvant targeted therapy is not yet significantly effective in improving the risk of all‐cause death (HR = 0.99, 95% CI 0.70–1.40).

The heterogeneity was moderate for included studies with an *I* ^*2*^ of 68%. After dividing all studies into two groups according to disease stage, the heterogeneity within the group was completely eliminated (*I* ^*2*^ = 0% in both groups). Therefore, disease stage was considered to be the main cause for the different OS benefits from adjuvant EGFR‐TKIs among different studies.

#### OS subgroup analysis of different treatment duration

Li et al.'s study and the ADJUVANT study were excluded from analysis due to lack of data on median medication time. The median medication time of the remaining two studies was extracted while the weighted average time was 22.2 months based on the sample size. Taking 22.2 months as the cut‐off value, the two studies were divided into two groups (Figure 4(b)).

For patients with a median treatment time over 22.2 months, the risk of death from all causes was significantly reduced. The HR for all‐cause death using EGFR‐TKIs was only 0.17 (95% CI 0.05–0.58). In studies with a median treatment duration less than 22.2 months, EGFR‐TKIs did not significantly improve the risk of all‐cause death (HR 0.46, 95% CI 0.07–2.90). Finally, the heterogeneity between the groups was high (*I* ^*2*^ = 85%, *p* < 0.01), which indicated that longer treatment duration was associated with better OS benefits.

#### OS subgroup analysis according to different comparation

According to the subgroup analysis based on different comparation (Figure 4(c)), the risk of all‐cause death of patients who received adjuvant targeted therapy after surgery was not significantly different from adjuvant chemotherapy (HR = 0.44, 95% CI 0.08–2.45) as well as placebo (HR = 0.70, 95% CI 0.25–1.98).

### Severe adverse events

**FIGURE 5:**
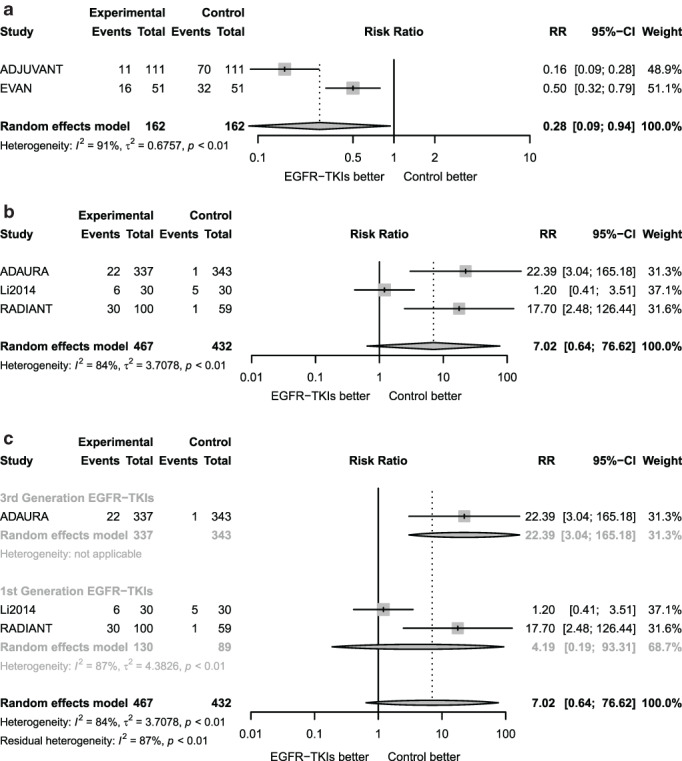
Forest plots of relative risk of severe adverse events. (a) Forest plot of risk ratio of severe adverse events associated with adjuvant EGFR‐TKIs versus chemotherapy. (b) Forest plot of risk ratio of severe adverse events associated with adjuvant EGFR‐TKIs versus placebo. (c) Subgroup analysis of risk ratio of severe adverse events for adjuvant EGFR‐TKIs versus placebo according to EGFR TKI type

For analysis on toxicity, adjuvant EGFR TKIs were shown to be significantly superior to chemotherapy and insignificantly inferior to placebo for rates of severe (grade 3 or higher) adverse events (RR = 0.28, 95% CI 0.09–0.94 and RR = 7.02, 95% CI 0.64–76.62, respectively; Figure 5(a),(b)).

To investigate possible reasons for heterogeneity, we did subgroup analyses with regard to the RRs by type of EGFR‐TKIs (third‐generation vs. first‐generation EGFR‐TKIs). As shown in Figure 5(c), heterogeneity between the two groups is extremely high (*I* ^*2*^ = 84%, *p* < 0.1), the risk of severe adverse events for third‐generation EGFR‐TKIs is higher than the placebo (RR = 22.39, 95% CI 3.04–165.18), while no difference was shown between first‐generation agents and the placebo (RR = 4.19, 95% CI 0.19–93.31). There was a significant increase in the risk of severe adverse events with third‐generation EGFR‐TKIs compared with first‐generation EGFR‐TKIs.

## DISCUSSION

In patients with NSCLC who have undergone radical surgery, adjuvant therapy is needed since micro metastases or residual tumor cells exist after resection. Previous meta‐analysis proved that adjuvant treatment with EGFR‐TKIs can reduce the probability of tumor recurrence and prolong DFS with fewer adverse events,^17^, ^20^ indicating that they can provide an alternative option to adjuvant chemotherapy in patients with EGFR‐mutated NSCLC.

Our study included the recently updated ADJUVANT and ADAURA studies, further confirming that adjuvant therapy with EGFR‐TKIs significantly improved DFS with a reduced recurrence risk of 62% in patients with stage IB–IIIA NSCLC who had undergone radical resection with positive EGFR mutations when compared with adjuvant chemotherapy or placebo treatment. This finding is consistent with the conclusions of previous meta‐analysis.^17^, ^18^, ^19^, ^20^, ^28^

With respect to OS, however, no significant difference was noted between adjuvant EGFR‐TKIs and chemotherapy/placebo. This finding may be explained by the usage of subsequent EGFR‐TKIs in the control arm after disease recurrence. In addition, OS was the secondary outcome in all included studies, so the sample size may be insufficient to detect difference in OS.^23^, ^24^, ^25^ Moreover, the OS data were not mature due to short follow‐up times with the exception of the ADJUVANT study, in which the median follow‐up time was 76.9 months. Therefore, more mature OS data are needed in the future to evaluate the impact of adjuvant‐targeted therapy on OS.

Disease stage is a prognosis factor for the therapeutic effect of adjuvant therapy. Previous literature reported that the median OS times were 37 months, 21 months and 9 months for NSCLC patients with lymph node staging as N0, N1, and N2, respectively.^29^, ^30^ In addition, whether stage IB patients can benefit from adjuvant therapy remained a question with controversies.^6^, ^31^ In the current study, the EVAN and Li et al.'s studies mainly focused on stage IIIA patients, while the ADJUVANT study included patients with stage II and IIIA disease. The ADAURA and RADIANT studies included patients with stage IB–IIIA NSCLC, and moreover stage IB patients accounted for as many as 47% of the overall population in the RADIANT study. Subgroup analysis showed that patients with stage IIIA disease got more benefit of DFS and OS from adjuvant EGFR‐TKIs compared with patients with heterogeneous stages less than stage IIIA. In the OS subgroup analysis, heterogeneity within the subgroups can be completely eliminated by division of the subgroups by different disease stages. Taken these findings together, patients with relatively later stages seem to benefit more from adjuvant EGFR‐TKIs.

Treatment duration was speculated to be related to the efficacy of adjuvant‐targeted therapy. Adjuvant treatment with 3‐year imatinib significantly prolonged DFS and OS compared to 1‐year imatinib in patients with operable gastrointestinal stromal tumor.^32^ EGFR‐TKIs yield a median progression‐free survival of 10–12 months in advanced NSCLC. Moreover, some patients continued to benefit from EGFR‐TKIs after tumor progression than switching to chemotherapy.^33^ In a phase II randomized 3‐month study with 2‐year adjuvant afatinib in surgically resected stage I–III NSCLC patients harboring EGFR mutation, 2‐year DFS was increased by 14% in patients treated for 2 years compared to those who were treated for 3 months (85% vs. 71%).^34^ However, this study was suspended due to slow enrollment, and the median DFS and OS had not yet been reached. Consistent with the findings mentioned above, our study found more DFS and OS benefits in patients with longer treatment duration. In the subgroup analysis for DFS, heterogeneity within the groups was completely eliminated when studies were divided according to the treatment duration, suggesting that the patients' DFS benefit from adjuvant EGFR‐TKIs depended on the medication duration to a great extent.

However, there was no relationship between treatment duration and OS benefit since only three studies were included in subgroup analysis. Additionally, two studies mainly included stage IIIA patients while 47% of patients in the other study had stage IB disease, so the heterogeneity between the two groups might be attributed to different disease stages rather than the length of treatment duration. More homogeneous studies are needed to investigate the relationship between treatment duration and OS benefit.

Our study first evaluated the relationship between DFS and EGFR mutation subtype. The 19 exon deletion and the 21 exon L858R point mutation are the most common dominant mutation subtypes of EGFR mutation.^35^, ^36^ Previous studies have shown there are significant differences in clinical characteristics between NSCLC patients harboring EGFR exon 19 deletion and exon 21 L858R point mutations.^37^ Patients with exon 19 deletion mutations also had longer progression‐free survival and higher objective remission rates than those with exon 21 L858R point mutations.^38^ However, no meta‐analysis has evaluated the efficacy of adjuvant EFDR‐TKIs in patients with these two common mutations so far. Subgroup analysis demonstrated that patients with exon 19 deletion mutations could obtain more DFS benefits from postoperative EGFR‐TKIs than those with exon 21 L858R point mutations. More research is needed to identify the underlying mechanism.

Emerging EGFR‐TKIs have entered clinical practice, including the first‐generation gefitinib, icotinib, and erlotinib, the second‐generation afatinib, and the third‐generation osimertinib. As a third‐generation EGFR‐TKI, osimertinib not only irreversibly inhibits sensitive mutations, but also effectively inhibits the T790M mutation that leads to drug resistance.^11^, ^13^, ^39^ Osimertinib was associated with significantly longer progression‐free survival (PFS) and OS than first‐generation gefitinib and erlotinib in patients with advanced NSCLC with EGFR mutations.^13^, ^40^ In the present study, postoperative third‐generation EGFR‐TKIs significantly reduced risk of disease recurrence when compared to first‐generation EGFR‐TKIs, providing a preferred option in the adjuvant setting. Furthermore, EGFR C797S mutation is one of the main mechanisms of resistance to osimertinib; to address this issue, the fourth‐generation EGFR inhibitor CH7233163 was developed.^41^

Despite the development of innovative adjuvant treatment with targeted therapy and immunotherapy, chemotherapy remains the cornerstone and standard therapy for adjuvant treatment. Meanwhile, adjuvant targeted therapy also faces many challenges, one of which is intratumoral heterogeneity. Lung cancer has been defined as a group of distinct diseases with molecular and phenotypic heterogeneity.^42^, ^43^ In spite of initial inhibition of cells with sensitive mutations, EGFR‐wild‐type clones emerge as the dominant source of resistance, leading to treatment failure. Several strategies have been developed to address this issue, including combination with chemotherapy and EGFR‐TKIs. In the first‐line setting, combining chemotherapy and EGFR‐TKIs have a significant impact on PFS for patients with advanced NSCLC.^44^ Therefore, it is worth exploring the role of adjuvant treatment with combined EGFR‐TKIs and chemotherapy. However, there is no direct comparison between combined adjuvant chemotherapy and EGFR‐TKIs and adjuvant EGFR‐TKI monotherapy. We performed subgroup analysis to investigate the relationship between DFS and previous chemotherapy before adjuvant EGFR‐TKIs, and found no significant difference. Because adjuvant chemotherapy itself could bring DFS and OS benefits to patients,^6^ it can be indirectly inferred that postoperative chemotherapy combined with adjuvant EGFR‐TKIs might bring more benefit to patients after surgery than using adjuvant EGFR‐TKIs alone. However, more studies are warranted to provide high‐level evidence with direct comparison, and the increased toxicity caused by combined therapy should be considered.

In the analysis of adverse events, we found that adjuvant EGFR‐TKIs were associated with fewer serious adverse events than adjuvant chemotherapy. Since patients with adjuvant EGFR‐TKIs have less risk of disease recurrence than adjuvant chemotherapy based on the results above, adjuvant EGFR‐TKIs could be a better choice for postoperative adjuvant treatment compared with adjuvant chemotherapy.

**FIGURE 6:**
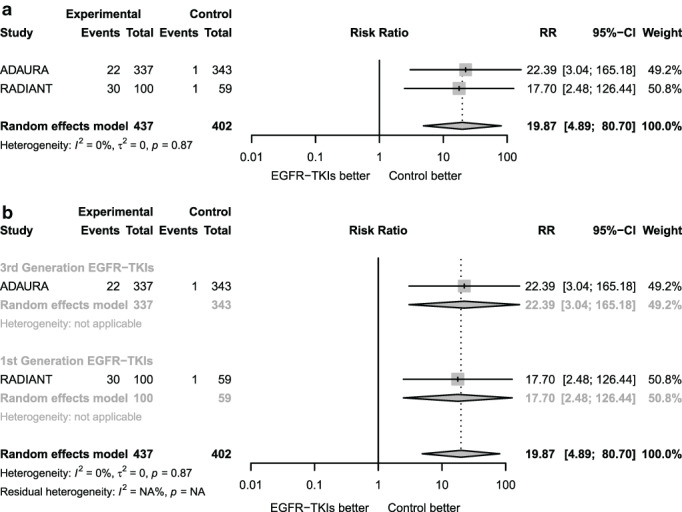
Forest plot for adverse events analysis excluding the study of Li et al. (a) Forest plot of risk ratio of severe adverse events for adjuvant EGFR‐TKIs versus placebo without the study of Li et al. (b) Forest plot on subgroup analysis of risk ratio of severe adverse events for adjuvant EGFR‐TKIs versus placebo according to different kind of drugs without the study of Li et al.

The present study also showed that the possibility of serious adverse events of third‐generation EGFR‐TKIs was significantly higher than that of first‐generation drugs, which was inconsistent with the results of previous study.^13^, ^40^ Looking back on the studies included in the analysis of adverse events comparing EGFR‐TKIs and placebo, the experiment design of Li et al. was different from that in the ADAURA and RADIANT studies. On the one hand, the treatment duration in the study of Li et al. was less than 6 months while it was over 20 months in ADAURA and RADIANT. Moreover, all the patients in the study of Li et al. received adjuvant chemotherapy previously while only some of the patients received adjuvant chemotherapy before EGFR‐TKIs or placebo treatment in ADAURA and RADIANT. Therefore, we excluded Li et al.'s study (Figure 6) and found that treatment with adjuvant EGFR‐TKIs was associated with more adverse events compared with the placebo. The possibility of severe adverse events for third‐generation drugs is similar to that for first‐generation drugs, which is consisted with the previous study.^14^, ^43^ The results for the adverse events for different drugs on adjuvant targeted therapy are therefore unstable and there is much variation between the results of the adverse event rates for first‐generation and third‐generation drugs in adjuvant targeted therapy.

This study was limited by the small number of included studies and the insufficient follow‐up time. There was only one study for third‐generation EGFR‐TKIs, which might result in bias to the subgroup analysis considering different drugs' efficacy. In addition, the experimental group, the control group, and the study population are quite heterogeneous. The ADJUVANT and EVAN studies were head‐to‐head studies comparing adjuvant EGFR‐TKIs and adjuvant chemotherapy, while the other studies implemented placebo as control. More importantly, we cannot conduct a more in‐depth subgroup analysis to explore the potential influencing factors since we failed to obtain patient‐level data. As a result, we could only perform subgroup analysis to discuss the benefit from adjuvant therapy according to stage indirectly without being able to analyze the data on patients in stage III and stage I–II separately.

## CONCLUSIONS

In summary, compared with adjuvant chemotherapy or placebo treatment, adjuvant EGFR‐TKIs significantly improved the DFS in EGFR‐mutant NSCLC patients with stage IB–IIIA disease who had undergone radical resection. However, no improvement of OS was seen. Advanced disease stages, exon 19 deletion mutations, longer treatment duration, and application of third‐generation EGFR‐TKIs were associated with more DFS benefits. Advanced disease stages and longer treatment time also had significant clinical implications for OS. Fewer adverse events were recorded in adjuvant EGFR‐TKIs than in chemotherapy. The risk of severe adverse events for first‐generation drugs is significantly lower than for third‐generation drugs. In the future, better‐designed and homogeneous research is needed to explore whether adjuvant EGFR‐TKI treatment will benefit patient OS and identify the specific patients who are suitable for and sensitive to this treatment.

## DISCLOSURE

No authors report any conflict of interest.
